# Metabolomics in infectious diseases and drug discovery

**DOI:** 10.1039/d1mo00017a

**Published:** 2021-04-12

**Authors:** Vivian Tounta, Yi Liu, Ashleigh Cheyne, Gerald Larrouy-Maumus

**Affiliations:** MRC Centre for Molecular Bacteriology and Infection, Department of Life Sciences, Faculty of Natural Sciences, Imperial College London London UK g.larrouy-maumus@imperial.ac.uk

## Abstract

Metabolomics has emerged as an invaluable tool that can be used along with genomics, transcriptomics and proteomics to understand host–pathogen interactions at small-molecule levels. Metabolomics has been used to study a variety of infectious diseases and applications. The most common application of metabolomics is for prognostic and diagnostic purposes, specifically the screening of disease-specific biomarkers by either NMR-based or mass spectrometry-based metabolomics. In addition, metabolomics is of great significance for the discovery of druggable metabolic enzymes and/or metabolic regulators through the use of state-of-the-art flux analysis, for example, *via* the elucidation of metabolic mechanisms. This review discusses the application of metabolomics technologies to biomarker screening, the discovery of drug targets in infectious diseases such as viral, bacterial and parasite infections and immunometabolomics, highlights the challenges associated with accessing metabolite compartmentalization and discusses the available tools for determining local metabolite concentrations.

## Abstract 2

Metabolomics has emerged as an invaluable tool that can be used along with genomics, transcriptomics and proteomics to understand host–pathogen interactions at small-molecule levels.

## Graphical abstract

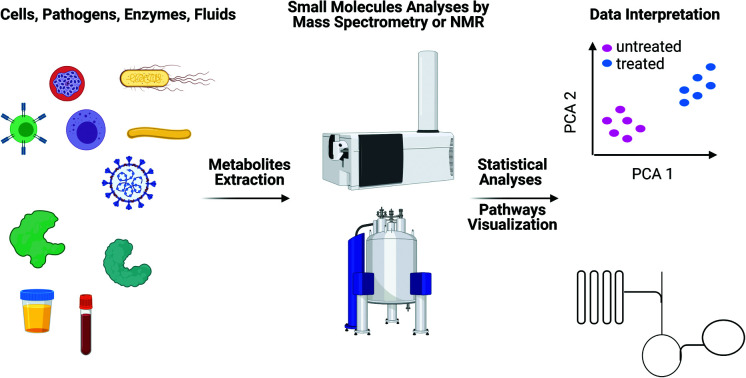

## Introduction

Metabolomics is a holistic approach towards the determination and quantification of metabolites in a biological system and the omics method most closely related to phenotype, last in the cascade of genomics, transcriptomics, and proteomics. Alterations at the metabolome level reflect disturbances in the preceding cascade, bridging the gap between the genome and phenotype. Metabolites are the products and intermediate molecules of metabolic pathways and include small molecules such as lipids, sugars, nucleotides, and amino acids. Changes at this level can precede the onset of disease symptoms, which renders metabolomics an essential diagnostic and prognostic tool crucial for investigating the mode of action of chemical compounds and obtaining an in-depth understanding of the impact of infection.

Two techniques are regularly employed for metabolomics analysis: mass spectrometry (MS) and nuclear magnetic resonance (NMR) spectroscopy. The main advantages of NMR lie in its non-destructive nature, reproducibility, and simple sample preparation, whereas its lack of sensitivity – limited to molecules at concentrations above 1 μM – poses the core limitation of this technique.^1,2^ Samples are placed in a magnetic field, and radio pulses alter nuclei with nonzero momentum. The spectra commonly used in NMR studies are ^1^H-NMR, ^13^C-NMR and ^31^P-NMR because these offer the highest sensitivity, and this sensitivity is further improved by advances in magnet technology and sample preparation – including cryogenically cooled probes and the introduction of separation methods such as liquid chromatography.^3–5^ Although NMR resonance ambiguity and overlapping signals can be improved by 2D experiments in which two spectra are recorded, the inherent high sensitivity of MS (picogram level) renders it ideal for biofluid analysis and one of the most widely used methods for metabolomics.

MS measures and separates molecules based on their mass-to-charge ratio (*m*/*z*) and requires three components: an ion source that generates ions, an analyser, and a detector. Different types of each component exist and are suitable for different experiments. MS analysis is usually preceded by the chromatographic separation of molecules, and this separation can be achieved by liquid chromatography (LC) and/or gas chromatography (GC), which are the most widely used techniques, as well as capillary electrophoresis (CE). LC/MS provides the highest coverage of the metabolome and includes hydrophilic interaction chromatography (HILIC) for polar metabolites and reverse-phase LC for molecules with higher hydrophobicity. Although the GC-MS coverage is lower than that of LC/MS, GC is ideal for volatile compounds.^6^ CE chromatography can also be used prior to MS analysis, provides medium coverage of the metabolome and is an ideal approach for charged metabolites.^7,8^

Metabolomics studies include two complementary approaches, targeted and untargeted metabolomics. The targeted approach is usually preceded by the formation of a hypothesis that needs to be tested and aims to quantify a predetermined set of metabolites.^9^ The untargeted method, which was initially called *fingerprinting*, is based on comparisons and the identification of differences between the complete metabolic profile of a reference and those of samples, *e.g.*, healthy and diseased individuals.^9^ The objective of untargeted metabolomics is to identify changes in metabolites caused by a specific factor under study, *e.g.*, an infection. Thus, the aim of this approach is not absolute quantification but rather the identification of differences between samples and the reference caused by a specific element in question, and the assembled list of altered metabolites can then be used for a targeted metabolomics study. Thus, untargeted and targeted metabolomics provides quantification of and insight into affected metabolic pathways. Metabolites are not unique to a single biochemical pathway, which makes their characterization and classification more complex than those of genes. For example, the Human Metabolome Database (HMDB, https://hmdb.ca/) and Metlin Database (hhttps://metlin.scripps.edu) are freely available electronic databases containing 114 264 entries (as of January 2020) and over 500 000 molecular standards, respectively.

One of the major bottlenecks of metabolomics is the generation of large volumes of raw data, which results in the need of multivariate statistical analysis in which each metabolite constitutes a variable.^10^ Principal component analysis (PCA) is an unsupervised method commonly used for initial visualization.^11^ The aims of data visualization are the identification of outliers and the determination of sample clustering. The difference in metabolites abundances can then be presented as fold-change differences between samples and controls, and the statistical significance of the differences is then measured such as by analysis of variance (ANOVA) and *T*-tests. Supervised methods require training on labelled data sets in which the outcome is known. Popular supervised methods include partial least squares regression discriminant analysis (PLS-DA) and orthogonal partial least squares regression discriminant analysis (OPLS-DA), which are associated with the risk of data overfitting,^10,12^ and thus, cross validation using techniques such as CV-ANOVA is essential for overcoming this issue.^13^

Metabolomics can provide a broad picture of the metabolome and insights into complex biochemical pathways by quantifying metabolites that are known players in major metabolic pathways, such as central carbon metabolism, including glycolysis, the tricarboxylic acid (TCA) cycle, and the pentose phosphate pathway. Glycolysis is the conversion of glucose to pyruvate and is used by proliferating cells because it generates adenosine 5′-triphosphate (ATP) while reducing nicotinamide adenine dinucleotide NADH to NAD^+^, which acts as a cofactor for many enzymes. The pentose phosphate pathway branches from glycolysis through the intermediate metabolite glucose-6-phosphate and diverts cell metabolism towards two outcomes: the synthesis of nucleotide and amino acid precursors for cell growth and proliferation and the synthesis of reducing metabolites for redox metabolism and fatty acid synthesis. The TCA cycle involves the conversion of pyruvate or fatty acids to multiple metabolites involved in ATP, NADH, and flavin adenine dinucleotide hydroquinone form (FADH_2_) production, the intermediates of which can be used for amino acid and lipid synthesis. NADH and FADH_2_ are used in mitochondrial oxidative phosphorylation (OXPHOS) metabolism to generate energy, which is used by nonproliferating cells to maintain their basal metabolism rates. Fatty acid oxidation involves the breakdown of fatty acids, is an alternative energy production pathway and is more effective for ATP generation than glycolysis. In contrast, fatty acid synthesis is used by proliferating and growing cells because it generates lipids that are required for maintaining the integrity of the cell structure. Moreover, amino acid metabolism is important for a wide range of metabolic pathways, such as protein synthesis, fatty acid synthesis, purine and pyrimidine synthesis.

Infection affects organisms in complex ways and often alters pathways involving enzymes encoded by uncharacterized genes. Activity-based metabolic profiling (ABMP) is a metabolomics approach that aids the functional annotation of genes by detecting subtle changes in metabolite abundances caused by recombinant enzyme expression.^14–16^ To gain further insights into the mechanistic changes to the metabolome in response to stimuli, metabolomics and, more particularly, stable isotope tracing analysis (*e.g.*, ^13^C, ^15^N, and ^2^H) allow a direct snapshot of pathway activities and metabolite regulation. Effectively, stable isotope labelling provides a unique picture of intracellular metabolism. Although untargeted and targeted metabolomics can provide the abundance of different metabolites within metabolic pathways, several metabolic changes do not *a priori* result in an increase or decrease in the metabolite level. Stable isotope tracing provides information not revealed by conventional untargeted metabolomics by measuring the rates of metabolite interconversion as a readout of metabolic enzyme regulation, which makes stable isotope tracer studies a powerful option for probing metabolic changes in complex biological systems. Insights into the full picture of cell metabolism obtained from the combination of targeted metabolomics and flux analysis data can inform biological research because answers from one platform can drive experiments on the other, resulting in a feedback loop for follow-up experiments.

Metabolism is central to the impact of infection on immunity because immune cells require the synthesis or degradation of different proteins, such as cytokine or cell surface receptors, to perform their different functions.^17^ A typical immune response begins with host exposure to a pathogen and initial infection, through several means such as inhaled aerosolised droplets or through the skin. The pathogen will then interact with their target cell of infection such as cells local to the area of infection or local innate immune cells such as tissue macrophages through host cell surface proteins called pattern recognition receptors. Once infected, these host antigen-presenting cells become activated and release signalling molecules such as cytokines, which can activate neighbouring cells. Within the antigen presenting cell, pathogen derived antigens bind to major histocompatibility complex (MHC) proteins and are presented at the cell surface to cells of the adaptive immune system to trigger an immune response specific to the invading pathogen. In the case of most viral and bacterial infections, the invading pathogen peptides, generated in the antigen presenting cell, will stimulate adaptive immune cells *via* MHC class I molecules, for viral infections and intracellular bacteria, or MHC class II molecules for extracellular bacterial infections.^18^ Activation of cytotoxic CD8^+^ T lymphocytes by MHC class I presentation of peptides leads to killing of the infected cells through a number of mechanisms. MHC class II with bound pathogenic peptide stimulates naive T lymphocytes to differentiate into activated T helper (Th1) cells and increase a specific immune response at the site of infection. These Th1 cells also aid in activating naive B cells to mature into plasma cells and initiate a humoral response through production of antibodies. In the case of parasites, the specific immune response to parasites leads to the production of antibody. Infection by protozoan parasites is associated with the production of immunoglobulin G (IgG) and immunoglobulin M (IgM).^19–21^ With helminths there is, in addition, the synthesis of substantial amounts of immunoglobulin E (IgE).^22^

In contrast, for parasitic infections, the innate immune response will instead stimulate an anti-inflammatory response through differentiation of T helper (Th2) cells and regulatory (Treg) T cells and the regulation and suppression of immune responses through cell surface receptors such as PD-1 and anti-inflammatory cytokines such as IL-10. These immunological processes are mirrored by a change in metabolism, which is dependent on the function and state of the differentiated cells. For example, during chronic viral infection, T cells can become exhausted as a result of continuous signalling through antigens or other immunostimulatory factors. Exhausted T cells form a distinct T cell population defined by the expression of inhibitory receptors (such as PD-1) and a decrease in effector functions compared with effector T cells and, importantly, exhibit a difference in metabolism.^23^ Exhausted CD8^+^ T cells are dependent on glycolysis during hepatitis B infection compared with effector cytomegalovirus-infected CD8^+^ T cells by upregulating the glucose transporter GLUT1 and are unable to carry out mitochondrial OXPHOS.^24^ In contrast, early infection with lymphocytic choriomeningitis virus (LCMV) suppresses both glycolysis and OXPHOS in the chronic strain clone 13 compared with an acute strain of LCMV called Armstrong,^25^ which highlights the differences in metabolism even between chronic viral pathogens.

As a result of this unique interplay between immune cell function and their metabolism, metabolomic studies of immune cells has become a research field with increasing popularity. Studies in immunometabolism – defined as metabolic alterations that affect immune cells – have mainly focused on the major metabolic pathways and their link to immune cell activation and function.^26^ These pathways include glycolysis, the TCA cycle, the pentose phosphate pathway, amino acid synthesis, fatty acid synthesis, and fatty acid oxidation.^26^ Altogether, these metabolic pathways are used by all immune cells at different stages of their development and in response to disease and are thus of great interest when attempting to understand the impact of infection on immunity. As such, the varying energy demands and functions of different cell types, such as macrophages and T cells, are directly linked to differential metabolic levels. The complex relationship between metabolic programming and cell phenotype offers the potential for therapeutic manipulation of the immune response.^27,28^

The study of the metabolome is the omics approach most closely related to the phenotype and is the most informative with respect to interactions between biological compounds. This review aims to highlight how metabolomics can be an invaluable tool for understanding host–pathogen interactions in infectious disease research by providing insights into its application for the discovery of biomarkers that can be used for disease diagnosis and its uses in drug discovery. We will further focus on the uses of activity-based metabolic profiling and how metabolomic flux provides a more complete picture. Topics such as immunometabolism and the emerging field of single-cell imaging metabolomics will also be introduced to showcase the versatility of metabolomic approaches in infectious diseases and the challenges faced.

## Metabolomics for host–pathogens interactions and biomarker discovery

Metabolomics analyses can be used to understand these host–pathogens interactions and screen host samples for biomarkers that are characteristic of a specific state. Those biomarkers can then be used for disease diagnosis, prognosis, and staging and for the assessment of new drugs with applications in viral, bacterial, and parasitic infections.

### Metabolomics and viral infections

The host innate immune response to several viral infections has been well studied. The immunometabolic response to the *Herpesviridae* family of viruses is the most studied; however, the response to many other viruses, such as Epstein–Barr virus, influenza virus, rhinoviruses, *Flaviviridae*, hepatitis B and C viruses, human immunodeficiency virus, and, more recently, SARS-CoV-2, has also been researched^29–31^ It is well established that during viral infections, the host metabolic response is manipulated by the virus to adapt to the increased virion replication rate by switching host cells from energy-producing metabolism, including aerobic glycolysis, to a more efficient means of energy production through anaerobic glycolysis.^29^

More recently, however, progress in understanding the role of inflammation and inflammatory metabolites and lipids on viral infectious diseases has been achieved. A substantial study of dengue virus infection was performed to investigate the metabolome (through LC/MS and GC/MS) and lipidome (through LC/MS/MS) of serum samples from 44 dengue virus-infected patients compared to 50 healthy control patients at three stages of the disease: less than 72 hours after presenting with fever, days 4–7, and weeks 3–4.^32^ The study identified several pathways, including fatty acid biosynthesis, fatty acid beta-oxidation, polyunsaturated phospholipid hydrolysis, lipolysis, and glycolysis, that were upregulated during the acute stages of dengue fever (the first two time points) and then returned to the control levels by weeks 3–4. The authors identified different polyunsaturated phospholipids that both promote and inhibit inflammation, which indicated that the host cell must balance the degree of inflammation during the infection to control the virus but not damage the host.

Several chronic viral pathogens infect T cells, and many are known to manipulate or suppress adaptive immune cells.^29^ In contrast to previous research, many recent studies have investigated changes in bioenergetics by using Seahorse technology to measure glycolysis and mitochondrial OXPHOS, the pathways for glucose breakdown and energy generation, while a few studies have used mass spectrometry technologies. Seahorse technology analyses the bioenergetics of live cells by measuring the oxygen consumption rate (OCR) and extracellular acidification rate (ECAR) and thus provides insight into cellular respiration and glycolysis.

### Viral infections – biomarkers

As biomarkers are metabolites whose abundance can be used as indicators of specific disease states or stages, they have been. They have been used to diagnose and evaluate progression of viral infections. These alterations in abundance that correlate to disease are often the result of the host immune response and the dysregulation of the main biochemical pathways in response to infection. This review will focus on four viral infections that have attracted numerous metabolomics studies and are highly relevant such as Human immunodeficiency virus (HIV), hepatitis B virus (HBV), hepatitis C virus (HCV) and severe acute respiratory syndrome coronavirus 2 (SARS-CoV-2).

### Metabolomics in HIV disease

Human immunodeficiency virus (HIV) is a retrovirus that causes acquired immunodeficiency syndrome (AIDS), which remains a global health issue. Due to substantial progress in HIV diagnosis and treatment, affected individuals can live with chronic infection while undergoing antiretroviral therapy (ART) despite the lack of a cure. Human immunodeficiency virus type 1 (HIV-1) is responsible for the majority of global AIDS cases, whereas only 30% of type 2 (HIV-2) infections develop AIDS.^33^ After infection, the HIV viral load reaches a peak at an excess of 1 million HIV RNA copies per mL after approximately two weeks.^34^ The acute and primary infection is followed by an asymptomatic stage that can last multiple years before symptoms arise and the disease progresses. Even during the asymptomatic phase, infection leads to gradual decrease in CD4^+^ T-cells, as the virus binds and infects the cells to replicate within them.^35^ HIV-2 exhibits lower virulence and transmission compared to HIV-1 and is described by slower CD4^+^ T-cell decline.

Metabolomics has been a versatile tool in HIV research and has been applied towards vaccine development and disease diagnosis. Studies have attempted to identify biomarkers from biofluids, such as plasma, that correspond to the protection afforded by potential vaccines. Initial efforts established that discrimination between HIV^+^ and HIV^−^ was possible by a comparison of the metabolic profiles of the serum of patients.^36^ A more interesting finding was the discrimination between HIV^+^ individuals who had received antiretroviral therapy (ART^+^) and HIV^−^ individuals,^36,37^ which was based on significant changes in the glucose and lipid levels. These findings were validated by Cassol *et al.* by untargeted ultrahigh-performance liquid chromatography UHLC/MS/MS and GC/MS of plasma and cerebrospinal fluid (CSF).^38^ The alterations discovered in HIV^+^ ART^+^ samples suggested an effect similar to accelerated ageing and involved neurotransmitters (glutamate, *N*-acetylaspartate), *myo*-inositol and ketone bodies. The identified metabolites were also some of the top-ranked classifiers for the development of HIV-associated neurocognitive disorders (HAND), and the results thus provide insight into the inflammation and neurotoxicity involved.

To characterize the factor responsible for the lower pathogenicity of HIV-2, HIV-1 and HIV-2 infections were compared based on their metabolic profiles obtained by LC/MS.^39^ Despite similar glycolytic and TCA profiles, the HIV-2 profile was characterized by an increase in deoxynucleotide triphosphate (dNTPs), which are hypothesized to be connected to HIV-2 viral protein x (Vpx). Vpx has been implicated in the degradation of SAMHD1, a host antiviral factor with deoxynucleoside triphosphate triphosphohydrolase (dNTPase) activity that aims to deplete dNTP availability for viral reverse transcription.^39,40^ Noninfected and HIV-infected primary monocyte-derived macrophages were used to extract metabolites for LC-MS/MS. Increases in the glycolysis intermediates fructose 1,6-bisphosphate (FBP) and glyceraldehyde 3-phosphate (G3P) were discovered with the HIV-1 strains, although the most remarkable change was the increase in quinolinate obtained with HIV-2 infections. Quinolinate is an upstream metabolite of NAD^+^ production in the kynurenine pathway, which starts with tryptophan degradation. Despite the observed changes in the quinolinate levels, NAD^+^ was not significantly diminished. Impaired function of the kynurenine pathway has been associated with various disorders, such as neurodegenerative diseases and chronic inflammation. In addition, the tryptophan levels have been linked to the immune response, and the continued depletion of this amino acid has been connected to T cell exhaustion and tryptophan catabolism toward immune activation.^41,42^ These results led to the speculation that the difference between HIV-1 and HIV-2 pathogenicity can potentially be attributed to tryptophan levels.^39^

Metabolomic studies using biofluids, such as urine, whole blood and serum, have also been employed to identify metabolite markers correlating to HIV-induced oxidative stress (OS).^43^ Studies using various methods (NMR, LC/MS, GC/MS, UPLC/MS), including both untargeted and targeted metabolomics, have explored changes indicative of OS, such as altered amino acid metabolism, *e.g.*, alanine and glutamine. Bipath *et al.* performed GC/MS analysis on 105 plasma samples from HIV^+^ sub-Saharan populations using a DB-5 MS capillary column and found increased levels of indoleamine 2,3-dioxygenase (IDO) in the HIV^+^ samples. This increase resulted in the upregulated breakdown of tryptophan and the accumulation of kynurenine pathway intermediates such as quinolinate and metabolites with neurotoxic properties compared with the results obtained with HIV^−^ and HIV^+^ samples from higher-income countries. These results reinforce the findings reported by Cassol *et al.*, who showed how tryptophan levels can be connected to inflammation and the development of HIV.^38,44^

### Application of metabolomics to hepatitis B

Metabolomics can greatly impact hepatitis B virus (HBV) research by providing a sensitive method for determining the stage of the disease without the need for high-risk methods such as biopsies and histology.^45^ After acute infection develops into chronic disease, the next steps are liver fibrosis (LF) and cirrhosis, which can progress to hepatocellular carcinoma (HCC).^45^

In 2015, Gao *et al.* used an untargeted GC/TOF workflow to identify metabolite markers that could be used to discriminate between HBV stages and support the early diagnosis of HCC based on 201 serum samples of various disease stages and healthy controls.^46^ These researchers successfully identified metabolites characteristic of HBV infection, progression to cirrhosis and perturbations towards discrimination between cirrhosis and HCC (asparagine and β-glutamate). Core pathways such as glycolysis and the TCA cycle undergo significant changes to enable HCC development; a blockage of the TCA cycle thought to be caused by observed increases in malic acid, citric acid, and succinic acid results in a dependence on glycolysis. They further proposed phenylalanine, malic acid and 5-methoxytryptamine as potential biomarkers for discrimination between HBV and controls and palmitic acid for cirrhosis recognition against HBV. In 2016, Shoeman *et al.* performed UPLC/MS analyses of 69 HBV positive and 19 control serum samples to study the metabolome during chronic HBV and further demonstrated how metabolic reprogramming can indicate disease staging.^47^ Similar to Gao *et al.*, these researchers detected increased ornithine levels in addition to increased levels of citrulline and glutamate, which pointed towards dysregulation of the urea cycle associated with liver damage.^47,48^ Furthermore, they proposed that the virus hijacks the glycerol-3-phosphate NADH shuttle to allow its replication, which offers a novel possibility for therapeutic intervention.

### Application of metabolomics to hepatitis C

Hepatitis C virus (HCV) is a leading cause of infection that can develop into chronic disease in 70% of infected individuals (WHO, 2018). The stages of fibrosis are often classified using the scale named METAVIR as F0, F1–2, F3 and F4, which range from no signs of fibrosis to cirrhosis, and a major risk of the latter stages is further development into HCC.^49^ Anti-HCV treatment has progressed substantially in recent years, and direct-acting antivirals (DAAs) have replaced pegylated interferon and ribavirin and offer effective treatment.^50^ Because metabolomics provides a link between genotype and phenotype, it has served as a useful tool in HCV diagnosis and disease staging.

Biomarkers for disease diagnosis have been identified using NMR, and a combination of MS coupled to different chromatographic methods have identified alterations in sugar metabolism and increased metabolites such as glucose in plasma.^51,52^ As in HBV, metabolomics can provide a sensitive and non-invasive method for disease staging, and many related studies have been performed. One of these attempted to use statistical methods to generate an algorithm to discriminate between stages by using amino acid ratios in plasma based on the formula [(phenylalanine)/(valine) + (threonine + methionine + ornithine)/(proline + glycine)] was generated using data mining and multivariate statistical analysis.^53^ Several links between the recent SARS-CoV-2 virus infections in patients with diabetes, and other metabolic disorders have suggested that immunometabolism plays an important role in infection. Plasma samples from 53 patients were analysed and their fibrosis state was determined using biopsies. Results showed that the formula could accurately discriminate between F3–F4 and earlier stages, as well as identify F4 against all other stages. Performance was measured by the area under the receiver operator curve, and results yielded high confidence (95% confidence interval). While these results are preliminary, they hold the potential for non-invasive liver fibrosis evaluation. Another study employed LC/MS and GC/MS and identified alterations that could be used as markers for fibrosis, *e.g.*, cysteine and bile acids.^54^

NMR has also proven useful in disease staging. An ^1^H-NMR approach used metabolite changes to distinguish serum from F0 and F4 patients, whereas the new DAA treatment enabled the study of a more representative metabolomic patient profile.^55^ Because DAA treatment directly targets viral replication and does not include biologically active molecules such as the previously used pegylated interferon (Peg-IFN), serum NMR spectra of 67 patients with HCV, 50 with HBV and 43 healthy controls were collected to characterize their metabolic fingerprints.^55^ Two one-dimensional ^1^H-NMR spectra of each serum sample were collected: one Nuclear Overhauser Effect (NOESY) spectrum, which shows protons close in space even if not bonded, and one Carr–Purcell–Meiboom–Gill (CPMG) train of pulses to enhance the signal.^56,57^ Differences between METAVIR levels were identified, and these focused on increases in the levels of tyrosine and formate, which are involved in multiple pathways, such as nitrogen and pyruvate metabolism. These results agreed with those obtained in previous studies and thus indicate the potential of using these molecules as fibrosis biomarkers.^58,59^ Meoni *et al.* also showed how DAA treatment reversed the changes to metabolite levels caused by infection and effectively differentiated between patients with HCV or HBV and healthy controls.^55^ HCV infection resulted in an increase in certain metabolites (lactate, 3-hydroxybutyrate, acetate, and pyruvate), indicating upregulation of the glycolysis pathway, which has been hypothesized to be induced by the virus. Similar to the results found for HBV, HCV diagnosis and staging can greatly benefit from the application of metabolomics.

### Metabolomics and SARS-CoV-2

The outbreak and rapid spread of SARS-CoV-2 in December 2019 has led to an unprecedented global pandemic causing over 100 million confirmed cases of COVID-19 (as of March 2021) and over 2.5 million deaths (WHO, 2021). The absence of an effective treatment renders its rapid and sensitive diagnosis a necessity. The speed and ease that metabolomics offers make it an excellent tool for the fight against COVID-19 because this approach can generate massive amounts of data and allows the rapid screening of molecules for the discovery of biomarkers for the diagnosis and prediction of disease severity.

Several links among the recent SARS-CoV-2 virus, diabetes, and other metabolic disorders have suggested that immunometabolism plays an important role in infection,^60^ but few studies have directly addressed these findings. One group compared whole blood metabolites from 17 SARS-CoV-2-positive patient samples with 25 SARS-CoV-2-negative healthy control samples using both NMR and LC/MS.^30^ The authors found several markers of inflammation in samples from patients with COVID-19: increased alpha-1-*cis* glycoprotein signal A, an increased kynurenine/tryptophan ratio, and the modulation of several lipid profiles, including high density and low-density lipoproteins and an increase in triglycerides. However, there is some discrepancy in their findings because the authors state that the kynurenine/tryptophan ratio is significantly higher in patients with COVID-19 according to their PCA and OPSL-DA analysis but was also found to be significantly lower in patients with COVID-19 based on the abundance values and the application of a Kruskal–Wallis rank sum test. Another study integrated several omics datasets (including metabolomics *via* GC-MS and AEX LC-MS/MS, proteomics *via* NanoLC/MS/MS, lipidomics *via* LC/MS, and transcriptomics *via* RNA-seq) from the blood of 102 patients with COVID-19 (including ICU and non-ICU-admitted patients) compared with 26 non-COVID-19 patients (including ICU and non-ICU-admitted control patients).^31^ An increased kynurenine/tryptophan ratio in patients with COVID-19 was not described in the study itself, but further investigation using their web app found that this ratio is higher in patients with COVID-19 in this dataset. Furthermore, the authors also identified an increase in triglycerides, which further supported the initial findings reported by Kimhofer *et al.*, and a decrease in several metabolites that are linked to decreased inflammation, such as citrate.^31^ While this study can confirm changes found in other analyses, the inclusion of ICU patients can identify which immunometabolomic pathways, in this study and others, are specific to SARS-CoV2 infection. These initial studies agree with several lines of evidence suggesting that COVID-19 stimulates a severe inflammatory reaction in the host and that patients with diabetes or cardiovascular diseases are at greater risk of severe COVID-19.^32,61^ These findings have helped identify underlying molecules promoting inflammation and other comorbidities in COVID-19 disease.

In addition to the immunometabolomic studies on COVID-19, there have been several studies identifying host biomarkers of COVID-19 diseases. A recent study showed that COVID-19-positive plasma samples were readily distinguishable from healthy controls by comparing the arginine/kynurenine ratios.^62^ Direct injection LC/MS/MS and ^1^H-NMR approaches were employed to measure 183 metabolites from plasma samples of suspected COVID-19 cases that were admitted to intensive care unit. These were later confirmed as cases (COVID-19^+^) or as negative (COVID-19^−^). A third group of age and sex matched healthy individuals acted as controls. The results showed a unique profile for COVID-19^+^, which was characterized by altered levels of kynurenine, creatinine, arginine, sarcosine and lysophosphatidylcholines. The kynurenine pathway starts with tryptophan degradation and results in the generation of energy in the form of NAD^+^. Increased levels of kynurenine point towards an increased degradation of tryptophan associated with the release of interferon-γ from activated T cells. The significance of arginine reduction could be attributed to its role in tissue repair. A significant step was also the identification of creatinine as a potential biomarker for disease severity; creatinine or the ratio of arginine to creatinine yielded 100% accuracy in the prediction of mortality. While the study included a limited number of test subjects, 10 in each group, the results exhibited 100% classification accuracy when distinguishing between COVID-19^+^ and controls, and 98% when distinguishing between COVID-19^+^ and COVID-19^−^. This underlines the potential of metabolomics for COVID-19 diagnostic uses, bypassing the need for polymerase chain reaction tests, and even more significant is the potential for disease severity prediction which could anticipate treatment requirements for each case.

Shen *et al.* used a combination of targeted proteomics and metabolomics to further test the hypothesis that serum from healthy individuals could be distinguished from that of infected individuals based on the metabolic profile.^63^ Alterations in 204 metabolites, including amino acids and carbohydrates, were detected by UPLC/MS/MS, and these can potentially serve as biomarkers for the assessment of case severity. Affected functions such as platelet degranulation and macrophage activity were also identified. The results were based on sera from 46 COVID^+^ subjects and 53 controls. They proposed a machine learning model for predicting cases that may become severe, which they trained on 31 patients of ranging disease severity. The model was validated on two test cohorts, where 7 out of 10 cases and 16 out 19 were correctly classified. While a higher number of clinical specimens and absolute quantification of metabolomic and proteomic data would be required before real-life application of these findings, the results remain promising. Another study performed LC-HRMS analyses of plasma of 55 infected individuals and 45 controls to identify markers for diagnosis with high accuracy and sensitivity.^64^ The multivariate model described in the study predicted SARS-CoV-2 diagnosis with accuracy, sensitivity and specificity greater than 74%. These researchers also demonstrated a link between the tryptophan-nicotinamide pathway and inflammation and potential implications of cytosine.

An attractive attribute of metabolomics approaches is the potential for the use of saliva, an easily collected, readily available sample that can be collected without the need for invasive methods. SARS-CoV-2 enters the organism *via* epithelial cell ACE2 receptors of salivary glands and the oral cavity, which makes saliva an attractive target for further metabolomics analysis that could potentially provide rapid, sensitive and accurate diagnosis.

### Metabolomics and bacterial infections

Several studies have used metabolomics to understand bacterial infections, typically focusing on the main cell type involved in the immune response to the majority of these infections: macrophage cells. Much information has been learned about bacterial infection through the stimulation of macrophage cells with lipopolysaccharide (LPS), a bacterial antigen that acts as a Toll-like-receptor 4 (TLR-4) ligand to activate downstream immune responses^65^ and thus acts as a pathogen-associated molecular pattern to stimulate immune cells such as macrophages towards an inflammatory phenotype. Before infection, resting macrophages exhibit low levels of metabolic activity but do carry out ATP generation through OXPHOS.^66^ Upon infection, however, different stimuli can induce macrophages to differentiate into what is broadly grouped as proinflammatory (M1) or anti-inflammatory (M2) macrophages. As the names suggest, proinflammatory cytokines *e.g.* IFNγ stimulates the differentiation of naïve macrophages into M1 macrophages, whereas anti-inflammatory antigens such as IL-4 stimulate the differentiation of naïve macrophages into M2 macrophages.^26^ The differentiation into these states has also been linked to different metabolic activities: M1 macrophages upregulate glycolysis and the pentose phosphate pathway, and M2 macrophages upregulate the TCA cycle and OXPHOS.^26^

M1 macrophages exhibit increased glycolysis activity while having a “broken” TCA cycle, which, upon LPS stimulation, synthesizes metabolites such as itaconate, a known antimicrobial molecule.^17^ A flux balance analysis study revealed that itaconate is the most abundant metabolite produced by bone marrow-derived macrophages (BMDMs) after LPS and IFNγ stimulation towards an M1 phenotype and reduces the secretion of proinflammatory cytokines such as IL-1β.^67^

In contrast, LPS stimulation has been linked to the production of other proinflammatory cytokines when host cells are stimulated with succinate. The synergistic effects of succinate and LPS increase IL-1β secretion from BMDMs through the production of reactive oxygen species and an increase in glycolysis.^68^ The same study also identified further roles for LPS and succinate in inflammation, including inhibition of the immune-suppressing cytokine IL-10. In addition, an HP-LC/MS analysis showed that LPS enhances the effects of IFNγ, an inflammatory cytokine, in BMDMs to increase the production of proinflammatory metabolites such as l-glutamate and (*S*)-malate.^17^ These reports demonstrate the diverse effects that one bacterial antigen, LPS, can have on the host immune response. Furthermore, the complex metabolic regulation by specific metabolites, such as itaconic acid and succinate, should be further studied using *in vivo* models to fully elucidate their roles during infection.

### Bacterial infections – biomarkers

Building on these studies focussing on the metabolomics of the host immune response, several host biomarker signatures have been discovered for the diagnosis, prognosis, and disease staging of bacterial infections.^8^ There are a variety of metabolomics technologies and host samples, like urine and blood plasma, used for the discovery of these biomarkers, leading to potential signatures to take forward for development into diagnostic tests applied to bacterial infections.

### Application of metabolomics to *Clostridium difficile*

*Clostridium difficile*, a Gram-positive bacterium, is a nosocomial pathogen that infects the gastrointestinal tract and causes potentially severe and highly recurrent disease. Biomarker discovery for the diagnosis and prognosis of recurrence has been an area of considerable interest. In 2016, both Allegretti *et al.* and Kao *et al.* identified metabolic changes for discriminating between healthy controls and infected patients. Allegretti *et al.* performed LC/MS analyses with an ACQUITY UPLC BEH C18 column on stool samples of 20 infected patients, 19 patients suffering from recurrent infection and 21 controls. They identified changes in bile salts that could differentiate between infection, recurrent infection, and healthy states^69^ and also proposed that the deoxycholate/(glycoursodeoxycholate + deoxycholate) ratio has the potential to be used as a biomarker for distinguishing primary and recurrent infections.^69^ Kao *et al.* performed NMR studies on urine of 31 infected subjects (age- and sex-matched to 31 healthy controls) and detected 53 metabolites, and which choline appeared to be the most relevant for the diagnosis of *Clostridium difficile* infection, possibly due to the absence of choline-metabolizing microorganisms.^70^ Similarly to Allegretti *et al.*, they demonstrated that discrimination between primary and recurrent infection was possible using histidine, a metabolite linked to *Clostridium difficile* infection, and *trans*-aconitic acid, whose role in infection remains unknown.^70^ In 2018, Zhou *et al.* performed UPLC/MS analyses of faecal samples for *Clostridium difficile* diagnosis and were able to detect obvious metabolome characteristics based on the abundance of molecules such as capsiamide and tyrosine.^71^

### Application of metabolomics to Tuberculosis

Tuberculosis (TB) is a bacterial, potentially lethal disease caused by the pathogen *Mycobacterium tuberculosis* (*Mtb*). While treatable, TB constitutes one of the top causes of death due to a single infectious agent (WHO, 2019). Despite estimates that show that ¼ of the world's population has been infected by *Mtb*, only ∼10% of these develop symptoms and active TB, whereas the rest maintain a latent form of the disease. HIV-infected individuals are particularly susceptible to TB progression. TB treatment lasts 6 months and involves a cocktail of antibiotics. The development of multiple drug-resistant and extensive drug-resistant (XDR) TB poses rising threats.

*Mtb* infection has been a particularly well-studied area of immunometabolism research because factors involved in *Mtb* metabolism and host cell metabolism have been linked to virulence and persistence during infection.^72^*Mtb* predominantly infects macrophages; therefore, macrophage metabolism has been extensively studied in the last decade.^73–75^ A balance of proinflammatory and anti-inflammatory responses is optimal for host control of *Mtb*; a recent study investigated the transcriptome and the metabolome of the two main macrophage subtypes in *Mtb* infection (alveolar and interstitial macrophages) to establish whether these represent the M1 or M2 phenotypes.^76,77^ Using several reporter *Mtb* strains, the authors demonstrated that alveolar macrophages presented with an M2 phenotype and were permissive to bacterial replication, whereas interstitial macrophages exhibited the M1 phenotype and showed reduced bacterial replication. Interstitial macrophages were found to produce a higher abundance of lactate than alveolar macrophages, as demonstrated by a lactate colorimetric/fluorometric assay, and were reduced in number upon inhibition with the drug 2-deoxy-d-glucose (2-DG), which suggested that higher glycolytic metabolism is beneficial to the control of bacterial replication,^77^ in agreement with previous studies.^78^ Furthermore, the inhibition of glycolysis in BMDMs using 2-DG increased bacterial growth as measured through colony forming units (CFUs), whereas the inhibition of fatty acid oxidation using the drug etomoxir reduced bacterial growth, which supported previous findings showing that glycolysis is critical for bacterial control.^77^

A recent comprehensive study used a more global approach to investigate *Mtb* infection in murine lung tissue over a time course of 9 weeks and compared LC/MS, GC/MS and CE/MS technologies.^79^ The authors identified previously known changes in innate immune cells, such as an induction of succinate metabolism as mentioned previously, and also identified changes in several metabolites involved in redox and oxidative stress pathways, including the inducible nitric oxide synthase (iNOS) and xanthine oxidase-related metabolites. Xanthine and hypoxanthine were decreased in the lungs of *Mtb*-infected mice 4 weeks post-infection, but their levels were increased by 9 weeks post-infection, which suggested the differential regulation of redox metabolites throughout the infection time course. iNOS metabolites arginine and citrulline were increased at both 4 and 9 weeks post-infection, which indicated an increase in iNOS, but the authors caution the interpretation of these results because it is unclear whether the increase in arginase originates from host cells or mycobacterial cells. This finding reflects the main limitation of the study; it is impossible to distinguish which metabolites are produced by the bacterial cell or the host cell and thus the specific host cell type in the lung. Nevertheless, this study provides the first time-course comparison of several metabolomic technologies investigating *Mtb* infection.

The host response to Mtb infection is an important field of study, not only because the role of metabolism in both Mtb and the host is critical for infection progression and outcome, but also for the development of diagnostic biomarkers for disease. While diagnostic tests exist for adult TB, albeit with poor sensitivity, there is currently no effective diagnostic test for paediatric TB.^80^

Most metabolomic efforts have aimed to provide faster and more sensitive methods for TB diagnosis and the identification of different types. Untargeted GC/MS has been used to compare metabolic profiles of sputum samples of healthy controls and patients with TB. The patients and controls formed identifiable clusters in PCA plots, and significant metabolite changes were noted in a variety of compounds, such as carbohydrates.^81^ Targeted and untargeted LC/MS studies of plasma and serum also revealed metabolic alterations that enabled discrimination between TB-positive patients and controls.^82–85^ Isa *et al.* made substantial progress toward TB diagnostics with their proposal of four urinary metabolites as biomarkers for active TB: diacetylspermine, neopterin, sialic acid, and *N*-acetylhexosamine.^86^ Using an untargeted HPLC-MS approach for the study of 102 urine samples from infected individuals, these researchers initially identified 49 significantly altered metabolites between TB cases and controls. This list was narrowed down to 10 metabolites using the area under the receiver operator curve (>85%), and four of these metabolites were identified by MS/MS. These results were further investigated using a blinded validation cohort of 50 people and longitudinal cohort of 20 that was followed during treatment. The results confirmed that the initial ten molecules formed a signature for active TB and thus provide a novel and non-invasive diagnostic approach. The majority of the metabolites – neopterin, kynurenine, spermine, *N*-acetylated sugars and sialic acids – are derived from the host and involved in immune cell activation.^87,88^

Metabolite markers can also be used as a prognostic tool. Weiner *et al.* studied metabolite perturbations in serum and plasma for the prediction of TB onset using 4462 HIV-negative study participants from East, West and South Africa.^84^ These subjects were household contacts of diagnosed TB cases. Their progress was followed over a period of 2 years after exposure to TB to monitor whether they developed active disease or remained healthy. The objective was the identification of a biosignature that could be used to predict which of them would develop active TB. The generated model made predictions using external and blinded datasets with relatively high specificity (75%) and sensitivity (69%). Some of the metabolites that were significantly altered included cortisol, mannose and amino acids such as histidine, cysteine, phenylalanine, and tryptophan. The identification of progressors to disease can allow early therapeutic intervention and control the spreading of the disease. TB is a characteristic example in which the diagnostic potential of metabolomics can be applied. Significant steps have been made towards the identification of a biosignature that could revolutionize diagnosis and facilitate timely treatment.

### Metabolomics and antiparasitic approaches

#### Application of metabolomics to malaria

Malaria is a life-threatening but preventable disease carried by female Anopheles mosquitoes. It is caused by intracellular parasites of hepatocytes and erythrocytes of the genus *Plasmodium*, of which *P. falciparum* and *P. vivax* pose the highest risk. According to the WHO, malaria caused more than 400 000 deaths in 2018 (WHO, 2019).

Metabolic markers for aiding non-invasive disease diagnosis, in addition to the prognosis of disease severity, have been an area of interest. MS has been applied to the identification of *P. falciparum* infection in plasma, and significant differences in metabolic profiles have been observed based on compounds such as amino acids and lipids.^89^ In 2015, Surowiec *et al.* used GC/MS to discern disease stages in paediatric infections by testing plasma from 421 individuals.^90^ The marked differences in severe cases of malaria included increased levels of 3-hydroxybutyric acid and fatty acids, which most likely derived from the host, and decreased levels of alanine and pyruvate, which are possibly connected to gluconeogenesis. A possible link between case severity and valine increase was also proposed and is conceivably connected to the digestion of haemoglobin.

Potential urinary biomarkers indicating active *P. falciparum* infection were also identified in a case-control study of 21 infected individuals and 25 controls using high performance liquid chromatography-high resolution mass spectrometry (HPLC/HRMS).^91^ The observed increases in metabolite levels were abated after antimalarial treatment. The altered levels of 1,3-diacetylpropane, *N*-acetylputrescine and *N*-acetylspermidine between patients and controls make these attractive biomarker candidates. The observed differences could be attributed to the “active phase responses”, which refers to the changes directly caused to the host by infection and parasite activity or to the host's response to infection, and these findings were consistent with the abnormal levels of amino acids and their metabolites (*e.g.*, threonine and trimethyl-l-lysine) found by Surowiec *et al.*^90,91^ A contradictory discovery was the levels of alanine, which appeared to be increased and support an enhanced glycolysis pathway. While most discoveries were supported in findings of preceding studies, the limited study subjects mean these results should be considered preliminary and in need of further validation using other datasets. The increased levels of urea linked to kidney injury were also of interest.^91^

Malaria is the cause of a significant global health burden, and metabolomics can aid non-invasive disease diagnosis and prediction of disease severity. The studies conducted to date have demonstrated that metabolic reprogramming caused by infection affects the gluconeogenesis pathway and supports kidney injury. The significance of multiple alterations remains unclear, which highlights the need for further experiments and the potential for future breakthroughs.

### Limitations and challenges

The study of metabolomics of the host immune response to infection can provide meaningful insights into global pathway differentiation, while its implementation in biomarker discovery is a valuable tool in the study of infectious diseases with applications in disease diagnosis, staging and assessment of treatment efficacy.

However, technical and biological challenges remain. MS and NMR each have distinct disadvantages, namely, a potential lack of reproducibility and sensitivity, respectively. Although most studies have focused on one approach, the combination of both could overcome the inherent limitations of each approach. Additional limitations are posed by the expensive nature of the instruments, which need to be operated by professionals. Targeted and untargeted metabolomics can detect a wide range of metabolites and link them to affected biochemical pathways, taking into consideration the challenge of metabolite identification. Furthermore, certain changes might not cause detectable perturbations in metabolite levels, and for the exploration of these changes, stable isotope tracing can potentially be used to provide insights into complex biological systems.

Challenges in downstream analysis include the lack of complete databases or metabolite standards available to characterize metabolites through techniques such as untargeted metabolomics. Often the bottleneck of a biomarker search is not the detection of metabolites, but the validation of their identity. In certain model organisms or diseases there is a need to improve upon current databases which can be done through comparison and integration of already existing tools to extract metabolite information^92^ and continued characterisation and validation of metabolites through further molecular studies.

In addition to the technical considerations, there is a growing number of immunometabolomics studies analysing infectious diseases which have used different models of infection. For example, studies focusing on respiratory pathogens such as *Mtb* have used a range of models, such as macrophage cell lines^79^ and whole lung tissue.^93^ Although this progresses our understanding of the disease, the use of multiple models with differing representations of true infection can lead to difficulty in understanding which of the results are replicated in *in vivo* infection. Furthermore, in certain multicell type models, bioinformatics analysis tools must be advanced to provide a more sophisticated interpretation of these complex datasets because we are currently unable to untangle from which cell each metabolite is derived.

Understanding the limitations of metabolomic studies is an essential step towards their resolution. The metabolome is affected by various external factors, like environmental and genetic conditions. A wide and diverse subject pool is essential for quality data that correspond to reality, in addition to optimised experimental design to reduce confounding variables. As evidenced in studies described earlier, different studies can result in inconsistent and sometimes contrasting results. Comparison of data can prove problematic due to the variation in analytical platforms, samples and their preparation in addition to subsequent data analysis. Collaborative initiatives where different laboratories can share techniques and results could aid in the standardization of operating procedures and could allow comparison between results and conclusions.

While metabolomics has inherent challenges to overcome in their application, their potential in disease diagnosis has been evidenced in the various studies reported earlier. An exciting future prospect for metabolomics is also their employment towards personalized or precision medicine.^94^ Since the metabolome reflects all variations of an individual's genomic, transcriptomic, and proteomic profile it would allow for the most efficient and accurate personalized treatment plan for both infectious and genetic diseases.

## Discovery of drug targets and deciphering of metabolome regulations

### Activity-based metabolomic profiling

Advances in sequencing technologies allow a more in-depth understanding of pathogens at the genetic level; however, our knowledge remains limited, with an estimated 30–50% of genetic sequences lacking functional annotation or having misannotations.^95,96^ The annotation of unknown genes is the current challenge in genomics and indicates the needs to better understand the differential physiology of pathogens and to identify potential targets of treatment. A large proportion of unannotated genes are expected to encode metabolic enzymes that could be fundamental to the survival and virulence of pathogens.^16,96^ Conventional genetic annotation is mainly based on *in silico* homology search and structural predictions, which is an approach that is extremely hard to use for genetic sequences lacking similarity to known genes.^16^

**Fig. 1:**
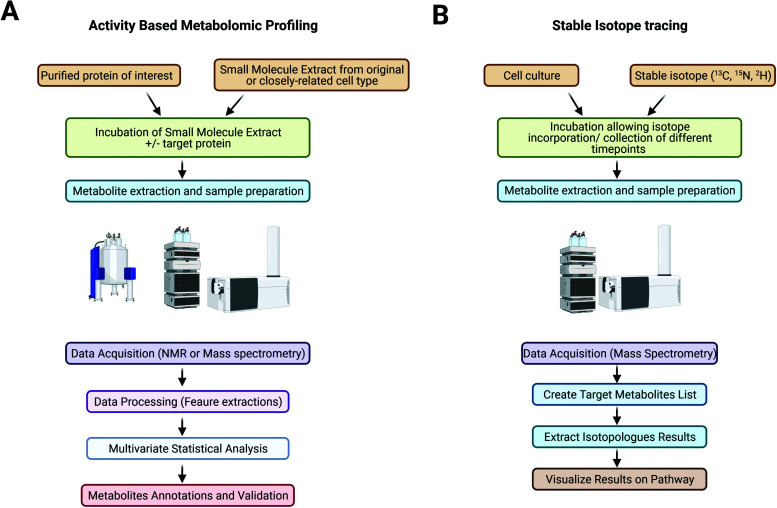
Workflow used to address the activity of an enzyme of unknown function by using activity based metabolomic profiling (A) and workflow used for stable isotope tracing analysis on an LC/QToF mass spectrometer (B).

The metabolome represents a pool of metabolites, including unknown metabolites, metabolites for which less knowledge of their structure and/or enzymatic kinetics is available, or less commercially available metabolites, which could be the substrate(s) of the enzyme of interest.^16^ Cell metabolite extracts can be prepared in large quantities, and the use of MS analyses enables the detection of subtle changes in metabolite abundances and thus allows a simpler profiling protocol that is efficient and accurate. Using the metabolome of the host organism or similar species also ensures the presence of putative cofactors and a nearly native environment for the enzyme to be studied. In contrast to traditional genetic studies, a genetically modified organism is not needed in this case, which also allows studies of the candidate protein that could be essential for growth. To set up an ABMP experiment, a recombinant enzyme of interest can be prepared, purified and incubated with the cellular metabolome extract from the same or closely related organism. The samples are then analysed by NMR or mass spectrometry coupled to a separation technique, such as LC/MS or GC/MS. By comparing the metabolomic signatures of the samples before and after target enzyme incubation, peaks indicating native substrates showing a decrease in abundance and products with an increase in abundance can be identified and annotated^16^ (Fig. 1A).

The general approach of metabolomic profiling using metabolome extracts was first performed by Saito *et al.*, who combined purified target enzymes from *E. coli* genes and metabolite pools from different sources.^97^ These researchers used CE-MS to analyse the mixture and identified two uncharacterized proteins, YbhA and YbiV that exhibit both phosphotransferase and phosphatase activities with different substrates. In another study by Saito *et al.*, a novel hydroxybutyrate dehydrogenase in *E. coli*, YihU, was annotated using similar approaches.^97^

De Carvalho *et al.* used the small metabolite extract of *Mycobacterium bovis* as a substrate library and incubated this extract with recombinant purified Rv1248c, a protein from *Mtb* that was previously annotated as a thiamine diphosphate (ThDP)-dependent alpha-ketoglutarate decarboxylase. By performing untargeted metabolomics analyses using high-throughput and accurate LC-MS methodology, a time-dependent consumption of alpha-ketoglutarate and the accumulation of 5-hydroxylevulinic acid (HLA) were identified and linked to the activity of the enzyme. Later studies confirmed the reaction using ^1^H-NMR spectroscopy, and Rv1248c was reannotated as a 2-hydroxy-3-oxoadipate synthase. The protein was also found to be essential for *Mtb* growth, and this finding highlights the ability of ABMP to assign functions to essential genes, which cannot be achieved by knockout mutant techniques.^14^ Similar ABMP techniques were also used to annotate mycobacterial protein Rv1692, a d,l-glycerol 3-phosphate phosphatase involved in glycerophospholipid recycling,^15^ and Rv3722c, the primary aspartate aminotransferase with essential roles in nitrogen metabolism and virulence.^98^ ABMP was also used by Lee *et al.* to identify the involvement of the methylcitrate cycle and glutamate synthase GltB/D in the adaptive metabolism of *M. bovis* BCG, which is different from the results obtained for *Mtb* and identifies a potential target for treatment of *M. bovis* BCG infection.^99^

Although most studies in the literature have studied mycobacteria, ABMPs can be applied to different organisms and show high potential in enzyme screening even in well-understood model organisms. Sevin *et al.* further developed the method by using cell lysate in addition to purified enzyme and combining it with database screening. This automated, high-throughput approach allowed the identification of 241 uncharacterized proteins in *E. coli* with putative enzyme functions, and 12 novel enzymes were validated in subsequent studies.^96^

ABMP can also be combined with multiomics techniques and structural studies to better study the properties of candidate enzymes in depth. For example, activity-based proteomic profiling (ABPP) is a technique that uses small activity-based molecule probes to identify proteins within a complex proteome and understand interactions between proteins and compounds.^100^ ABPP is being increasingly used to study uncharacterized enzymes and identify drug targets, but its limitations include a dependence on synthetic chemistry for the production of probes and the inability to identify native substrates of the target.^16^ A combination with metabolomic techniques, including ABMP, can improve the understanding of the target enzyme by allowing substrate identification in near-native conditions. Due to the development of automated protocols such as machine learning in data screening and qualification to improve the efficiency and accuracy, this technique shows high potential for enzyme annotation and drug target discovery.

### Metabolomic flux studies using stable isotope tracing

The metabolome represents a complicated network of reactions and pathways, and understanding the relative changes in the abundances of key metabolites does not represent the full map of the occurring events.^101,102^ Measurement of the metabolic flux, which is the rate of interconversion between metabolites reflecting the activities of enzymes and the whole pathway in response to different conditions, allows access to this information.^103,104^ By using stable isotope tracing techniques, the incorporation of heavy isotopes from nutrients into intracellular metabolites through a network of metabolic pathways could be measured and compared between conditions to reveal key pathways and potential targets for treatment.

Metabolic flux analysis (MFA) could be designed using stationary or nonstationary experiments. In conventional stationary labelling studies, the isotopic distribution is measured after a steady state is reached, which could take hours to days and require constant experimental conditions. The method is used to determine the relative contribution of one nutrient to the synthesis of metabolites but cannot be used to effectively target a single pathway or to capture transient changes in metabolic flux.^103^ On the other hand, isotopic nonstationary MFA measures changes in labelling profiles over time and thus provides more details on metabolic reactions.^104^ The method shortens the duration of the labelling treatment time but requires rapid sampling and quenching techniques to obtain an accurate dataset.

Stable isotopes (*e.g.*, ^13^C, ^15^N, ^2^H, and ^18^O), usually in the form of labelled nutrients, are supplied to cell culture (Fig. 1B). Depending on the purity of the isotope tracer, different aspects of metabolic pathways might be studied; for example, labelling with 50% but not 100% ^13^C-glucose allows identification of a differential labelling pattern in the carbon backbone of pyruvate that reflects the involvement of the pentose phosphate pathway.^105^ Tracers with heavy isotopes at specific positions might also be used to reveal complicated pathways.^103^ Metabolically quenched samples are processed and analysed by NMR or mass spectrometry.^101^ MS-based technologies are more widely used due to their high sensitivity and wide range of detection.^106^ GC/MS is more commonly used for the analysis of sugars, amino acids and fatty acids and can provide more positional information on heavy isotopes incorporated into metabolites,^104^ whereas LC/MS provides a wider range of detection for metabolites and the simultaneous quantification of metabolites and is thus more suitable for nonstationary MFA.^103^ Metabolites are annotated by species-specific databases based on *m*/*z*, retention times and isotopologue patterns, or further experiments such as MS/MS could be performed for the identification of isotopomer patterns. Changes in abundances and labelling patterns can be analysed using tools such as MassHunter Profinder (Agilent Technologies) and XCMS (https://xcmsonline.scripps.edu) a nonlinear alignment of liquid chromatography mass spectrometry data sets. Annotated metabolites can be mapped onto metabolic pathway network using online databases such as KEGG pathway (https://www.genome.jp/kegg/pathway.html) and MetaCyc (https://metacyc.org/), which also provide species-specific databases for more accurate annotation and details of related enzymes and other metabolites for further investigation. Other tools such as VistaFlux (Agilent technologies) and iPath 3 (https://pathways.embl.de/) allow import of LC-MS data for automatic generation of customisable pathway map and data visualisation in various forms. Table 1 displays the most common metabolites and metabolic pathways that can be found.

**Table 1:** Target metabolites list and their formula, representative of core metabolic pathways

Information pathways	Metabolite	Formula
Glycolysis	β-d-Glucose 6-phosphate	C_6_H_13_O_9_P
	d-Fructose 6-phosphate	C_6_H_13_O_9_P
	β-d-Fructose 1,6-bisphosphate	C_6_H_14_O_12_P_2_
	d-Glyceraldehyde 3-phosphate	C_3_H_7_O_6_P
	Dihydroxyacetone phosphate	C_3_H_7_O_6_P
	Pyruvic acid	C_3_H_4_O_4_
	Lactic acid	C_3_H_6_O_3_

Pentose phosphate pathway	d-Ribose-5-phosphate	C_5_H_11_O_8_P
	d-Gluconate-6-phosphate	C_6_H_13_O_10_P
	d-Ribulose-5-phosphate	C_5_H_11_O_8_P
	d-Gluconic acid	C_6_H_12_O_7_

Bioenergetics	NAD^+^	C_21_H_27_N_7_O_14_P_2_
	NADH	C_21_H_29_N_7_O_14_P_2_
	ADP	C_10_H_15_N_5_O_10_P_2_
	ATP	C_10_H_16_N_5_O_13_P_3_

TCA cycle	*cis*-Aconitic acid	C_6_H_6_O_6_
	d-Threo-isocitric acid	C_6_H_8_O_7_
	α-Ketoglutaric acid	C_5_H_6_O_5_
	Succinyl-CoA	C_25_H_40_N_7_O_19_P_3_S
	Acetyl-CoA	C_23_H_38_N_7_O_17_P_3_S
	Succinic acid	C_4_H_6_O_4_
	Fumaric acid	C_4_H_4_O_4_
	Malic acid	C_4_H_6_O_5_
	Oxaloacetic acid	C_4_H_4_O_5_

Urea cycle and nitrogen metabolism	Citrulline	C_6_H_13_N_3_O_3_
	l-Ornithine	C_5_H_12_N_2_O_2_
	Arginine	C_6_H_14_N_4_O_2_
	Arginosuccinic acid	C_10_H_18_N_4_O_6_
	Glutamine	C_5_H_10_N_2_O_3_
	Glutamic acid	C_5_H_9_NO_4_
	γ-Aminobutyric acid	C_4_H_9_NO_2_

Amino acids	Alanine	C_3_H_7_NO_2_
	Arginine	C_6_H_14_N_4_O_2_
	Asparagine	C_4_H_8_N_2_O_3_
	Aspartic acid	C_4_H_7_NO_4_
	Cysteine	C_3_H_7_NO_2_S
	Glutamine	C_5_H_10_N_2_O_3_
	Glutamic acid	C_5_H_9_NO_4_
	Glycine	C_2_H_5_NO_2_
	Histidine	C_6_H_9_N_3_O_2_
	Isoleucine	C_6_H_13_NO_2_
	Leucine	C_6_H_13_NO_2_
	Lysine	C_6_H_14_N_2_O_2_
	Methionine	C_5_H_11_NO_2_S
	Phenylalanine	C_9_H_11_NO_2_
	Proline	C_5_H_9_NO_2_
	Serine	C_3_H_7_NO_3_
	Threonine	C_4_H_9_NO_3_
	Tryptophan	C_11_H_12_N_2_O_2_
	Tyrosine	C_9_H_11_NO_3_
	Valine	C_5_H_11_NO_2_

Flux analysis techniques are powerful for studying the differential response of pathogens under varied environmental factors or in response to drug treatment. The functions of genes with putative roles can be studied using this method in addition to transcriptomics, which can provide a more complete understanding. One example is the cyclic AMP receptor protein (CRP), a global transcriptional regulator in *Mtb* with a regulon identified in genomic studies but whose downstream metabolomic events are not understood. By performing untargeted LC-MS analysis and ^13^C isotope labelling, its effects on nitrogen metabolism and peptidoglycan synthesis were revealed, and the results complemented the transcriptomics data.^107,108^ A comparison between metabolomic networks of closely related pathogens can also be achieved by stable isotope labelling to understand the functions of enzymes.^109,110^

The technique is also applied to investigate the effect of drug candidates and drug combinations for clinical treatment. Cobbold and McConville summarized a mass spectrometry-based protocol for detecting the effects of antimalarial drugs on *Plasmodium falciparum*, and through its coupling with stable isotope tracing, the method can provide a broader understanding of overall metabolomic perturbations.^111^ An earlier study by the same research group investigated the metabolic changes in *P. falciparum* and infected red blood cells upon treatment with a panel of clinical drugs and inhibitors.^112^ ^13^C-glucose stable isotope labelling was used for metabolite profiling with GC/LC-MS, and specific metabolic signatures after treatment with drugs including atovaquone, chloroquine and proguanil were identified. Moreover, a flux analysis showed that dihydroartemisinin (DHA) disrupts pyrimidine biosynthesis and haemoglobin catabolism, resulting in a redirection of the metabolic flux, which could explain the susceptibility of *P. falciparum* to DHA treatment during its early blood stage. This methodology can be applied to study the mode of action of novel antimalarial drugs and their overall impact on both parasite and host cells.

Another example is the study of the mode of action of bedaquiline (BDQ), a new antibiotic for TB. Upon primary inhibition of *Mtb* ATPase, BDQ triggers a complex set of secondary reactions that are less understood. Using ^15^N labelling, glutamine biosynthesis was found to be almost completely inhibited and largely correlated with a decrease in ATP levels after BDQ treatment. The major glutamine synthetase GlnA1 was found to be sensitive to changes in the ATP levels in response to BDQ treatment. Supplementation with methionine sulfoximine, a glutamine synthetase inhibitor, showed synergistic effects with BDQ with an 18-fold decrease in the minimal inhibitory concentration. This finding provides a new understanding of the mode of action of BDQ and the collateral vulnerability of *Mtb*, and the results could be applied to the design of new drug combinations for the treatment of drug-resistant TB.^113^

One limitation of *in vitro* stable isotope tracing is that the metabolomics of pathogens is largely affected by the environment, and the outcome of such studies could be less relevant to clinical conditions. The study of host–pathogen interactions could provide valuable insight into key events in the infection process and suggest potential targets for treatment. Recent studies applying stable isotope tracing methodologies on the host–pathogen interface include HIV–macrophage interactions during the neuropathogenesis of AIDS;^114^ membrane remodelling and utilization of host metabolites by a largely unknown glycerophosphocholine pathway during *Streptococci* infection;^115^ and measurement of the metabolism at the tachyzoite stage of *Toxoplasma gondii* separated from host cells.^116,117^

Stable isotope tracing and flux analysis can also be used to study host response to infection. T cells play important roles in adaptive immunity. Stimulated T cells differentiate into effector T cells, and during this process, the cellular metabolism is remodelled to support proper proliferation and immune function. Several studies have performed *in vitro* cultures and identified metabolites key for T cell function, including glutamine, acetate and arginine,^118–121^ but the differential use of nutrients by cells *in vivo* is less understood. Ma *et al.* used a combination of bioenergetics, proteomics and metabolomics with ^13^C stable isotope labelling techniques to study the nutrient use of T cells activated by *Listeria monocytogenes in vivo* and identified higher bioenergetic plasticity with an increased rate of oxidative metabolism. *In vivo* labelling experiments were performed by intravenously infusing [U-^13^C] glucose into anaesthetized, *L. monocytogenes*-infected mice followed by T cell isolation from the spleen, and subsequent analysis revealed that *in vivo* T effector cells displayed differential utilization of pyruvate into the TCA cycle preferentially through the pyruvate dehydrogenase complex instead of pyruvate carboxylase, which is preferentially used by *in vitro*-activated T effector cells. Additionally, a prominent flow of glucose carbon into anabolic metabolic pathways, including nucleotide and serine biosynthesis, was identified, and the latter was found to be essential for T effector cell proliferation. This study highlights the importance of investigating immune cell metabolism *in vivo*, and by using isotope labelling techniques, key differences between *in vivo* and *in vitro* T cells were identified. Understanding immune cell metabolism and nutrient regulation at different stages of T cell proliferation provides inspiration for immune system modulation upon infection.^118^

Another study by Mills *et al.* focused on the mode of action of itaconate, an endogenous macrophage regulatory metabolite found at increasing concentrations upon activation of macrophages by LPS.^122^ In addition to its ability to disrupt the activity of succinate dehydrogenase (SDH), this study found that itaconate alkylates cysteine residues on the protein KEAP1 and prevents downstream degradation of Nrf2, which in turn activates antioxidant and anti-inflammatory pathways. ^13^C-Glucose and ^13^C-glutamine were added to cells, entered the TCA cycle and were used to synthesize itaconate. In comparison to the control group, cells treated with LPS showed a higher rate of ^13^C incorporation into itaconate and a higher abundance of itaconate–cysteine adducts. In combination with tandem MS, which confirmed alkylation of cys151 on the KEAP1 peptide upon treatment with 4-ocyl itaconate (a permeable itaconate derivative), the authors were able to confirm the ability of itaconate to modify KEAP1. The researchers thus proposed a negative feedback loop in which LPS stimulates the production of itaconate and thus promotes an anti-inflammatory response *via* Nrf2 activation and SDH inhibition, which in turn downregulates its own production through the IFN pathway.^122^ Further understanding of itaconate and related pathways can provide new insights into the inflammatory response to infectious diseases and improve clinical outcomes.

Stable isotope labelling techniques are also increasingly used in drug development for studying the absorption, distribution, metabolism and excretion (ADME) of drugs, their toxicities and their activities against targets.^102,123^ The incorporation of stable isotopes into parent drugs or drug substrates will allow the monitoring of the metabolic flux in the system and the analysis of drug–target interactions with potentially affected pathways, which is important prior to the design of therapeutics, particularly in cases in which the host metabolism is largely altered, such as genetic disorders, cancer and inflammation.^102^^13^C and ^15^N are widely selected for these studies, whereas ^19^F is present in some classes of drugs that can also be targeted for MFA, mainly by NMR.^102,124,125^ Detection of the toxicity of reactive drug metabolites that react with cell proteins or DNA and result in adverse effects can also be achieved by stable isotope labelling and MS techniques. For example, glutathione (GSH) has been used for trapping reactive metabolites due to its natural scavenging properties, and ^13^C-labelled GSH has been used in combination with MS to detect glutathionylated drug conjugates.^126–128^

Based on the abovementioned literature, stable isotope labelling and MFA techniques are widely used to study both pathogens and hosts, as well as their interactions with drugs or drug combinations. Because advances in MS techniques provide higher resolution and wide coverages for the detecting of isotopologues of metabolites, the methodology acts as an invaluable tool with increasing importance in metabolomic studies. The choice of analytical platforms for stable isotope labelling studies is based on the natures of the samples and the types of metabolites of interest, and a combination of techniques, including GC/MS, LC/MS, tandem MS and NMR, could be applied to obtain a full coverage map of metabolic flux.^102^ Moreover, MFA requires metabolite databases with higher coverage and accuracy for species-specific metabolites, and the integration of databases in multiomics platforms with automated annotation features would accelerate the processes of metabolite identification and pathway building.

Future directions of metabolic flux studies include the identification of its spatial and temporal patterns using dynamic labelling experiments and imaging mass spectrometry, which could determine local concentration and location of intracellular metabolites, and machine learning-based flux analysis (MLFA) could be applied to a large set of samples by predicting flux ratios based on a small set of isotopologue measurements.^129^ Dynamic profiling of the metabolomic pathway can also be applied to pharmacometabolomics and personalized medicine by *in vivo* MFA, which is becoming important in cancer biology to monitor the tumour microenvironment, in allergen immunotherapy and in studies of diet and nutrition.^130–133^ Various factors affecting the treatment outcome for individuals, including levels of drug tolerance, ADME profiles of drugs and efficiencies of drug combinations, could be monitored precisely and used to build a personalized profile for disease prevention, early intervention and efficient, targeted treatment.

## Recent advances: single-cell metabolomics and imaging

Although metabolomics provides metabolic information on immune cells, linking metabolic pathways with other molecules, such as proteins, is important to fully understand the biological mechanisms of action of many metabolites. Multiomic integration studies would help link metabolites with other factors involved in immune pathways. A handful of these studies have been previously described,^31,65,67,77^ but the majority of multiomic analyses have focused on genomics, transcriptomics and proteomics. Most of the studies that incorporate metabolomics analyses do not fully integrate the data with another-omics dataset; instead, most simply use one dataset to separately confirm the other. Tools that can model relationships between datasets have been developed but have yet to be used in these studies. Although multiomic studies are beginning to be published, the scale, cost, and complexity of the data prevents most research groups from using this approach.

Mimicking the move in the field of transcriptomics, single-cell metabolomics is a recent development in immunometabolomics. Instead of analysing samples containing several cells and possibly several cell types that might provide conflicting results, single-cell metabolomics focuses on the metabolic profiles of individual cells. The major progress towards single-cell metabolomics originated from the development of tools to extract single cells from samples for analysis. These tools involve extracting metabolites from both single cells on a solid surface and in suspension and have been comprehensively reviewed.^93^ Despite multiple reviews on the topic, the literature on single-cell metabolomics focuses on the development of methods for this technique, and the few studies so far have investigated immune cells,^134^ cancer,^135^ and the microbiome.^93^ As this field develops further, we will hopefully see more advances in the field of immunometabolism in infectious disease.

The move towards studying individual cell metabolism is accompanied by studies aiming to discover the metabolism inside tissue or cellular compartments, which is known as metabolomics imaging and is a growing field that uses mass spectrometry-based technologies, mainly laser capture microdissection LC/MS (LCM-LC/MS) and matrix-assisted laser desorption/ionization mass spectrometry (MALDI-MS), to ‘image’ a cell or structure. Despite the apparent importance of immunometabolomics in disease, metabolomics imaging has focused predominantly on evaluating drug efficacy in specific disease models. For example, several models have investigated antibiotics in response to *Mtb* infection because *Mtb* is an intracellular pathogen that can form large cellular structures called granulomas in patient lungs, which might prevent the efficacy of certain drugs.^136^ One study using LCM-LC/MS confirmed that ethambutol, a first-line tuberculosis drug, accumulates at a dose suggested to be lethal to the bacteria in almost all granuloma layers in rabbits; however, the researchers did not measure the bacterial levels directly.^136^ Another study performed a similar LCM-LC/MS-based investigation for another first-line tuberculosis drug, pyrazinamide, in rabbit lung granulomas and found that the levels of the drug were similar in all granuloma layers as in rabbit plasma and that these concentrations were effective at lowering the bacterial load after 5 weeks of treatment.^137^ Furthermore, the authors also used another method, MALDI-MS, to analyse tuberculosis drugs in rabbits and several other animal models, including marmosets and mice.^137^ Although these studies are extremely relevant for the clinical implications of *Mtb* drug use, all focused on animal models, and their applicability to human infection is unclear. To the best of our knowledge, no studies have addressed immunometabolism from an immunology perspective, which has resulted in a gap in our understanding of metabolism in immune cell compartments and structures.

A recent remarkable application of metabolomic imaging was performed by Pareek *et al.*, who used high-resolution gas cluster ion beam secondary ion mass spectrometry (GCIB-SIMS) to image single cells and visualize the purinosome in action.^138^ Purinosomes catalyse the *de novo* synthesis of purines and consist of nine independent enzymes. Using a focused beam on a frozen layer of HeLa cells, MS spectra were collected three dimensionally at 1 μm resolution. By combining the spectra across all layers, a two-dimensional picture of the cell was constructed. This study highlighted how metabolomics can be used to probe single-cell biochemistry. After single-cell imaging, research interest also shifts to subcellular visualization of different compartments. Greenwood *et al.* applied this method to visualize the localization of BDQ in *Mtb*.^139^ Human monocyte-derived macrophages were infected with *Mtb*, treated with antibiotics, fixed and imaged by correlative electron microscopy and ion microscopy. The localization of BDQ was correlated with the signal of the bromine atom and was visualized as interacting with host lipid droplets. These researchers proposed a mechanism in which BDQ and possibly other lipophilic antibiotics collect in lipid droplets that interact with the bacteria and are consumed, which results in the enhancement of antimicrobial activity. The emergence of single-cell imaging metabolomics and applications in subcellular localization demonstrate the versatility of metabolomic approaches and show promise for future studies.

## Conclusions

This review aimed to provide a concise overview of the wide-ranging advances in metabolomics in recent years, the various applications of this technology in infectious diseases, drug discovery and immunology, and the recent advances in single-cell imaging. Metabolomics has classically been appreciated for its value in biomarker discovery, which can aid disease diagnosis and prognosis and assess the efficacy of therapeutic treatment or identify elements that confer protection against diseases. However, its importance is not limited to the screening of biomarkers; stable isotope tracing can provide unique insight into metabolic pathways, and ABMP is a rapid and accurate approach for assigning biochemical action to unknown enzymes. Immunometabolism links metabolic changes to immune cells and offers the potential for further understanding of infection and therapeutic interventions. Moreover, single-cell metabolomics has emerged as a powerful method for the spatial localization of metabolites and shows much promise for the future of the field. Altogether, the vast array of application of metabolomics shows great promise to both further our understanding and tackle infectious diseases.

## Author contributions

V. T., A. C., Y. L. and G. L. M. conceived the review and wrote the manuscript.

## Conflicts of interest

There are no conflict to declare.

## Supplementary Material
